# Adolescent Endometriosis: From Clinical Management to an Early Recognition and Prevention-Oriented Paradigm—A Narrative Review

**DOI:** 10.3390/healthcare14193241

**Published:** 2026-10-01

**Authors:** Adriana Ioana Gaia-Oltean, Teodora Telecan, Laura-Ancuta Pop, Miana Gabriela Pop, Alexandru Florin-Badea, Romeo Micu

**Affiliations:** 1 Faculty of Nursing and Health Sciences, Iuliu Hatieganu University of Medicine and Pharmacy, 400012 Cluj-Napoca, Romania; oltean_adriana_ioana@yahoo.com (A.I.G.-O.); romeomicu@hotmail.com (R.M.); 2 Department of Anatomy and Embryology, Iuliu Hatieganu University of Medicine and Pharmacy, 400012 Cluj-Napoca, Romania; afbadea84@gmail.com; 3 Department of Pathology, County Emergency Clinical Hospital, 400347 Cluj-Napoca, Romania; 4 Genomics Department, Medfuture-Institute for Biomedical Research, Iuliu Hatieganu University of Medicine and Pharmacy, 400012 Cluj-Napoca, Romania; laura.ancuta.pop@gmail.com; 5 Department of Public Health and Management, Faculty of Medicine, Iuliu Hatieganu University of Medicine and Pharmacy, 400012 Cluj-Napoca, Romania; dr.miana.hosu@gmail.com

**Keywords:** adolescent endometriosis, early-onset disease, dysmenorrhea, chronic pelvic pain, diagnostic delay, menstrual suppression, prevention-oriented care

## Abstract

Background: Adolescent endometriosis remains underrecognized, although symptoms often begin after menarche and diagnosis may be delayed for years. Severe dysmenorrhea, chronic or acyclic pelvic pain, school absenteeism, and impaired functioning should not be normalized. This narrative review synthesizes evidence on adolescent and early-onset endometriosis, focusing on early recognition, non-invasive assessment, individualized care, and function-preserving follow-up. Methods: A structured narrative review search supported by PubMed/MEDLINE was updated on 9 August 2026, focused mainly on the last decade, with selected older studies included when relevant. Adolescent-specific evidence was prioritized; adult or mixed-age data were used only when applicable to diagnosis, imaging, treatment safety, or pathophysiology. Evidence was synthesized descriptively; no meta-analysis, formal risk-of-bias assessment, or formal evidence grading was performed. Results: The synthesis included 58 unique publications. Direct adolescent evidence supports early suspicion in severe dysmenorrhea, refractory or acyclic pain, functional impairment, family history, and obstructive Müllerian anomalies. Imaging may identify endometriomas, deep endometriosis, adhesions, or adenomyosis-related features, but normal imaging does not exclude superficial disease. Biomarkers remain investigational in adolescents. Hormonal treatment is primarily supported for pain, bleeding, and functional outcomes. Conclusions: Early recognition, timely referral, expert imaging when appropriate, individualized treatment, and follow-up centered on functional impact may reduce symptom burden in adolescents. Hormonal suppression is a clinically plausible strategy for symptom control and preservation of functioning, but it is not a proven disease-modifying or fertility-preserving intervention in adolescents.

## 1. Background

Over the past two decades, endometriosis research has expanded considerably in adult populations; however, investigation into adolescent endometriosis is comparatively underdeveloped, leaving gaps in evidence-based diagnostic and treatment approaches [1,2,3,4,5,6,7,8,9].

Endometriosis diagnosis is commonly delayed by 5–8 years in adult women; however, in adolescents, the delay often extends beyond 10 years. This suggests that symptom onset or early disease expression may occur during the early postmenarchal period, when severe menstrual pain is frequently misclassified as primary dysmenorrhea [2,5,6,7,8,10]. In this review, adolescents refers primarily to postmenarchal girls and patients within the adolescent age range, while teenagers is used only as a non-technical synonym. Young women refers to older adolescent or young adult cohorts when studies did not isolate adolescent outcomes. Early-onset endometriosis refers to symptoms or diagnosis beginning during adolescence or early reproductive life. 

Endometriosis is commonly classified into three main phenotypes: superficial peritoneal endometriosis, ovarian endometrioma, and deep endometriosis [11,12]. In adolescents, symptom severity may not correlate with visible lesion burden, and normal imaging does not exclude superficial or early disease [7,11,13,14,15,16]. Therefore, the evaluation of suspected adolescent endometriosis should not focus only on lesion detection, but also on persistence, recurrence, response to initial treatment, and impact on school attendance, sleep, sport participation, social functioning, and quality of life [5,6,7,8,17,18]. Recent reviews and guidelines have addressed adolescent endometriosis, imaging, non-invasive diagnosis, and clinical management [2,3,7,8,9,12,13]. The added value of this review is to integrate these areas into a single clinically oriented framework that prioritizes disabling dysmenorrhea, functional impairment, expert imaging when appropriate, cautious interpretation of biomarkers, individualized treatment, and longitudinal follow-up. Evidence directly involving adolescents is distinguished from adult or mixed-age evidence, and mechanistic hypotheses are presented as biologically plausible rather than definitive proof of disease progression.

The objective of this, as a narrative review, supported by a structured PubMed/MEDLINE search, was to synthesize the available literature on adolescent and early-onset endometriosis, with emphasis on early clinical recognition, non-invasive assessment, treatment selection, safety considerations, selective surgery, and research gaps. The review was not designed as a systematic review or meta-analysis, and its conclusions should be interpreted as a narrative synthesis of heterogeneous evidence.

## 2. Materials and Methods

### 2.1. Review Design

This manuscript was designed as a narrative review supported by a structured PubMed/MEDLINE literature search, not as a systematic review or scoping review. The objective was to synthesize current evidence on adolescent and early-onset endometriosis, with emphasis on clinical presentation, diagnostic delay, risk factors, imaging, non-invasive diagnosis, medical management, treatment safety, surgical indications, recurrent pain, and prevention-oriented care. No formal SANRA score was calculated; however, the manuscript was prepared with attention to principles relevant to high-quality narrative reviews, including a clear rationale, transparent source selection, balanced synthesis, and explicit discussion of limitations, in line with the SANRA framework.

Evidence derived specifically from adolescents was prioritized. Publications were considered adolescent-specific when they focused on adolescents, teenage patients, postmenarchal girls, or adolescent/young female cohorts in which adolescent outcomes were directly discussed. Adult or mixed-age evidence was retained only when adolescent-specific evidence was limited and when the findings were clinically relevant to adolescent management, particularly for general pathophysiology, guideline recommendations, contraceptive eligibility, bone health, venous thromboembolism risk, treatment safety, imaging, or surgical care. These sources were interpreted as contextual evidence and were not considered equivalent to adolescent-specific prospective data. The flow diagram, eligibility criteria, and evidence table were included to improve transparency, not to imply systematic exhaustiveness.

### 2.2. Literature Search Strategy

A structured electronic literature search was performed in PubMed/MEDLINE, as detailed in Table 1. The final search update was performed on 9 August 2026. PubMed/MEDLINE was selected as the primary biomedical database because the review focused on clinical gynecological adolescent health, imaging, and guideline literature. The exclusive use of one database is acknowledged as a limitation and as a potential source of publication and selection bias. Manual screening of reference lists from relevant reviews, guidelines, and adolescent-focused studies was also performed to identify selected seminal or clinically relevant publications not captured by the primary date-limited search.

The following PubMed filters were applied: English language, humans, and publication date primarily from 1 January 2016 to 9 August 2026. No automatic female-population filter was applied when it risked excluding pediatric or adolescent records because of incomplete indexing. Selected pre-2016 publications were retained only when identified through manual reference screening and considered adolescent-specific, guideline-relevant, seminal, or clinically important for dysmenorrhea, imaging, treatment safety, bone health, pathophysiology, or surgical management.

### 2.3. Screening and Eligibility Process

**Figure 1:**
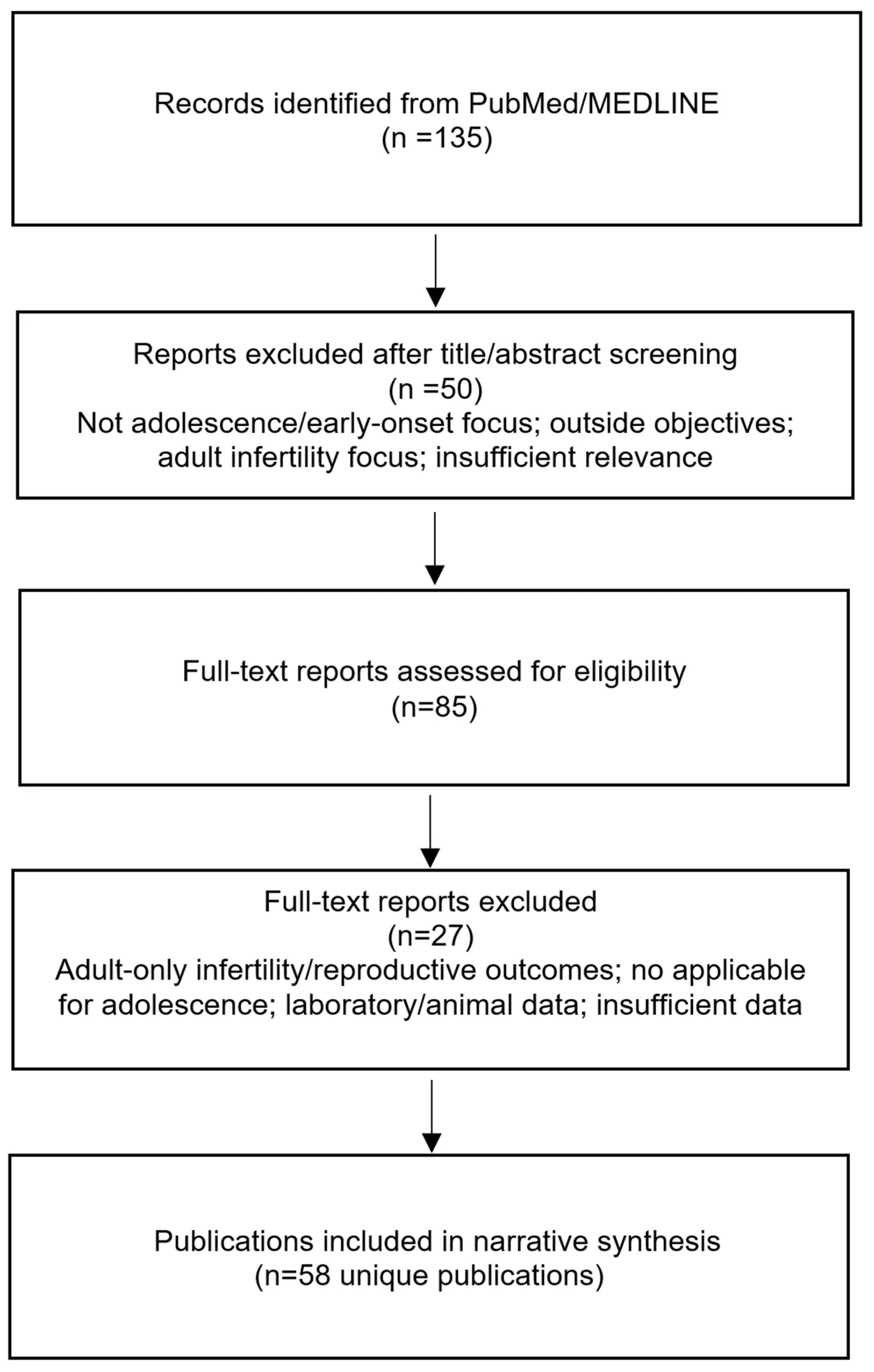
Flow diagram of the literature selection process.

The PubMed/MEDLINE search retrieved 135 records. During title and abstract screening, 50 records were excluded because they were not focused on adolescent or early-onset endometriosis, did not address the review objectives, focused on adult infertility or reproductive outcomes without adolescent relevance, or had insufficient relevance based on the available title and abstract. Therefore, 85 records remained for full-text eligibility assessment. Screening and eligibility assessment were performed by two authors and discussed jointly; disagreements were resolved by consensus. No third-reviewer adjudication or dedicated systematic-review screening platform was used. After full-text assessment, 27 reports were excluded because they focused on adult infertility or reproductive outcomes without adolescent relevance, did not directly address the review objectives, included adult or mixed-age data not applicable to adolescents, were laboratory or animal studies without clinical applicability, or provided insufficient clinical or methodological detail. The final narrative synthesis included 58 unique publications. The study selection process is summarized in Figure 1, and the principal characteristics of the included publications are presented in Table 2.

The diagram summarizes the structured PubMed/MEDLINE search, title and abstract screening, full-text eligibility assessment, reasons for exclusion, and final selection of publications included in this narrative review. It is provided to improve transparency and should not be interpreted as indicating that this manuscript is a systematic review.

### 2.4. Inclusion and Exclusion Criteria

Studies and publications were included if they addressed at least one of the following topics: adolescent or early-onset endometriosis; dysmenorrhea or chronic pelvic pain in adolescents; diagnostic delay; adolescent-specific clinical warning signs; family history; early menarche; obstructive Müllerian anomalies; ultrasound or magnetic resonance imaging; adenomyosis; non-invasive diagnosis; hormonal suppression; combined hormonal contraceptives; progestin therapy; dienogest; GnRH agonists or antagonists; add-back therapy; bone health; venous thromboembolism risk; surgical indications; central sensitization; quality of life; or biomarkers.

Adult or mixed-age studies were included only when their findings were clinically relevant to adolescent care and when adolescent-specific evidence was limited. These sources were used for contextual interpretation, pathophysiological background, guideline-based care, imaging, or safety counselling, not as direct proof of adolescent-specific outcomes. Studies were excluded if they were unrelated to adolescent or early-onset endometriosis, focused exclusively on adult infertility without relevance to adolescent management, were animal-only or laboratory-only studies without clinical applicability, were not available in English, or lacked sufficient clinical detail for narrative synthesis.

### 2.5. Data Extraction and Synthesis

For each included publication, the following information was extracted: first author, year of publication, study type, population or focus, whether the evidence was adolescent-specific or contextual, and the principal contribution to the review. Extracted evidence was organized into thematic domains: adolescent clinical presentation, diagnostic delay, dysmenorrhea and chronic pelvic pain, imaging, non-invasive diagnosis, risk factors, obstructive Müllerian anomalies, medical therapy, treatment safety, bone health, venous thromboembolism risk, surgery, and quality-of-life outcomes.

Because of substantial heterogeneity in study design, population, diagnostic criteria, treatment exposure, and outcome reporting, no pooled statistical analysis was performed. Findings were synthesized descriptively. Source type and applicability were reported and interpreted cautiously; however, no formal risk-of-bias assessment or evidence grading was undertaken, and expert opinion or mechanistic hypotheses were not presented as equivalent to direct adolescent clinical evidence.

### 2.6. Methodological Quality and Risk of Bias Assessment

A formal methodological quality assessment or riskofbias assessment was not performed, because this manuscript was designed as a narrative review rather than a systematic review or meta-analysis. Therefore, clinical statements should be interpreted as a narrative synthesis of available evidence and not as graded clinical recommendations. The absence of a formal riskofbias assessment and evidence grading represents a methodological limitation of this review. The strength and applicability of sources were considered narratively, with priority given to adolescent-specific studies, adolescent-focused reviews, international guidelines, and studies directly addressing adolescent diagnosis, treatment, imaging, safety, or surgical management.

## 3. Main Findings

The literature search identified 58 unique publications addressing adolescent endometriosis from complementary clinical, diagnostic, imaging, therapeutic, surgical, and safety-related perspectives. Evidence derived directly from adolescent populations was prioritized, while adult or mixed-age data were retained only when they provided relevant contextual information for pathophysiology, treatment safety, imaging, or guideline-based management.

### 3.1. Clinical Presentation, Suspicion Factors, and Adolescent-Specific Risk Framework

Endometriosis is estimated to affect approximately 10% of women of reproductive age [24]. The available literature consistently indicates that adolescent endometriosis remains underdiagnosed, even though symptoms frequently begin during the postmenarchal period [2,3,4,5,6,7,8]. The true prevalence in adolescents is difficult to establish because studies use different inclusion criteria, diagnostic pathways, and confirmation methods. Some cohorts are based on surgical diagnosis, whereas more recent studies increasingly incorporate expert ultrasound or magnetic resonance imaging [13,14,15,16,33]. This methodological heterogeneity partly explains the variability in reported prevalence and highlights the need for adolescent-specific diagnostic pathways [2,9,13].

Risk-factor assessment in adolescents should not rely exclusively on adult reproductive epidemiology [2,6,22,23]. Classical factors such as early menarche, short menstrual cycles, prolonged bleeding, family history, and low body mass index remain relevant, but adolescent evaluation requires additional emphasis on clinical red flags [5,6,17,20,21,22,23,26,27]. These include severe dysmenorrhea beginning at or shortly after menarche, poor response to NSAIDs or hormonal treatment, acyclic pelvic pain, school absenteeism, sleep disruption, limitation of daily activities, and obstructive Müllerian anomalies [6,7,8,17,28,29,35,36].

Obstructive Müllerian anomalies deserve particular attention in adolescents because impaired menstrual outflow may favor retrograde menstruation and early development of endometriosis [28,29]. Recent studies on obstructive reproductive tract anomalies support active evaluation for genital tract obstruction in adolescents with severe cyclic pain, especially when pain begins soon after menarche or is associated with progressive symptoms [28,29].

Current evidence suggests that heritable factors contribute approximately half of the overall risk of developing endometriosis, underscoring a substantial genetic component to its multifactorial etiology [2,4]. Important risk factors are listed in Table 3 and Table 4.

### 3.2. Diagnosis and Imaging

Several imaging-focused studies support the value of expert ultrasound evaluation in adolescents with severe dysmenorrhea [14,15,33]. In adolescents, the choice of ultrasound approach should consider clinical indication, sexual activity, patient preference, consent, comfort, and local expertise. Transabdominal, transrectal, transperineal, or transvaginal ultrasound may be considered according to acceptability and clinical context. Referral to clinicians experienced in adolescent gynecological imaging is justified when pain is severe, persistent, recurrent, functionally disabling, refractory to initial therapy, or associated with suspected deep endometriosis, adenomyosis, obstructive anomalies, adnexal masses, or complex anatomy [3,7,13,14,15,16,33].

Magnetic resonance imaging (MRI) may be particularly useful when ultrasound is inconclusive, when transvaginal examination is not acceptable, or when deep endometriosis, adenomyosis, or complex anatomy is suspected [11,13,16]. Studies assessing MRI in adolescent populations suggest that imaging can contribute to earlier identification of pelvic endometriosis, although MRI should not replace clinical judgment and should be interpreted in the context of symptoms, functional impairment, and response to treatment [3,13,16].

### 3.3. Conceptual Framework: Repetitive Ovulatory Menstruation, Inflammation, Pain and Early Intervention

Ovulation and menstruation are physiological events that involve tightly regulated inflammatory and tissue-remodeling processes [34,54]. In susceptible adolescents, repetitive ovulatory-menstrual activity may provide a biologically plausible context for pelvic inflammation, pain amplification, and possibly early endometriosis or adenomyosis-related changes; however, this remains a conceptual framework rather than proof of inevitable disease progression [10,34,54]. Menstruation involves progesterone withdrawal, vasoconstriction, ischemia, endometrial breakdown, bleeding, leukocyte recruitment, prostaglandin release, matrix degradation, and subsequent tissue repair [34]. Retrograde menstruation may transport viable endometrial fragments into the peritoneal cavity, but additional genetic, immunological, anatomical, and microenvironmental factors are likely required for lesion implantation and persistence [4,10,11,28,29,34].

**Figure 2:**
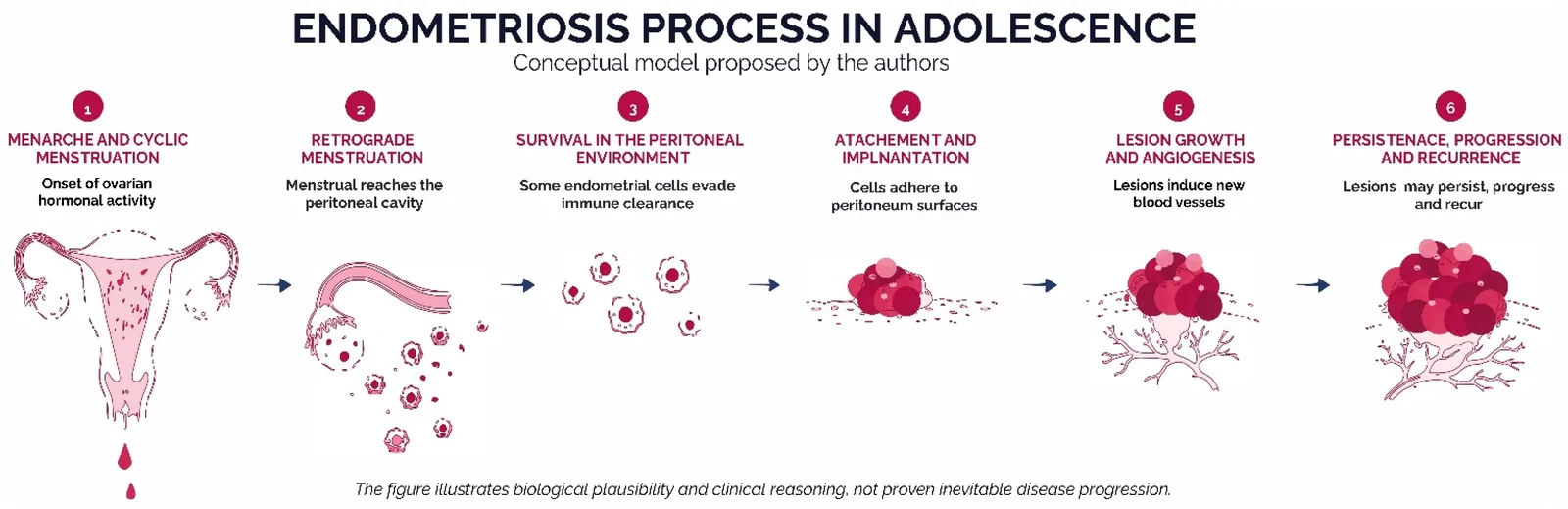
Conceptual model of endometriosis development in adolescence. The figure illustrates a plausible progression from early menstrual events to lesion establishment and recurrence. It represents biological plausibility and clinical reasoning, not proven inevitable disease progression. The figure was drawn using GStarCAD 2025 (Gstarsoft Co., Ltd., Suzhou, China) and Acrobat Pro 2026 (Adobe Inc., San Jose, CA, USA).

Figure 2 presents a hypothetical model proposed by the authors to illustrate biologically plausible links between early postmenarchal ovulatory-menstrual cycles, retrograde menstruation, survival of endometrial cells in the peritoneal environment, implantation, angiogenesis, lesion growth, and possible persistence or recurrence. It should not be interpreted as proof of inevitable disease progression.

This model also helps explain why adolescent lesions may be small but highly symptomatic. Early lesions may be metabolically active, vascularized, and neurogenic; therefore, symptom severity may not correlate linearly with lesion size or surgical stage [11,19,25].

Adenomyosis may follow a related but anatomically distinct process involving the endometrial–myometrial junction [16,33,34]. Repeated uterine contractions, menstrual tissue injury, local inflammation, and remodeling at the junctional zone may contribute to early myometrial changes [33,34]. In adolescents, mild ultrasound or MRI features suggestive of adenomyosis may coexist with endometriosis or severe dysmenorrhea, supporting the concept that early-onset endometriosis and adenomyosis may represent overlapping manifestations of repeated inflammatory menstrual activity in susceptible patients [15,16,33].

Recurrent dysmenorrhea during adolescence is not merely a repeated monthly symptom. In susceptible patients, repeated nociceptive input may contribute to peripheral and central sensitization, leading to amplification of pain, reduced pain thresholds, and the development of acyclic or persistent pelvic pain [8,10,25,37]. This may explain why some adolescents continue to experience substantial pain even when imaging findings are limited or after visible lesions have been treated [11,25,37].

Central sensitization should be considered when pain becomes less clearly linked to menstruation, when pain extends beyond the pelvis, when fatigue and sleep disturbance are prominent, or when symptoms overlap with bladder, bowel, musculoskeletal, or headache syndromes [8,25,37]. In these cases, disease-directed treatment alone may be insufficient, and management should include a broader pain-informed strategy [3,8,37]. The psychosocial dimension is also central. Adolescents with endometriosis-related pain may experience school absenteeism, impaired academic performance, reduced sport participation, social withdrawal, anxiety, depressive symptoms, and frustration related to delayed diagnosis [6,17,37]. These outcomes are not secondary concerns; they are part of the disease burden and should be included in clinical assessment [3,6,37].

For this reason, adolescent management should move from a purely lesion-based approach toward a function-preserving framework that documents pain intensity, analgesic use, bleeding pattern, school attendance, sleep quality, physical activity, psychological distress, treatment tolerability, and need for escalation of care [3,7,8,12,37].

### 3.4. Biomarkers and Non-Invasive Diagnostic Approaches

The diagnostic approach to endometriosis is evolving from mandatory surgical confirmation toward a combined model based on clinical suspicion, expert imaging, empirical treatment when appropriate, and selective use of emerging non-invasive tools [7,12,13,38,39]. In adolescents, diagnostic laparoscopy should not be the default first step for every patient with severe dysmenorrhea, particularly when no urgent surgical indication is present [7,12].

Epigenetic regulation, microRNA expression, inflammatory pathways, and immune dysfunction have become important areas of investigation in endometriosis [38,39]. Salivary microRNA signatures are promising non-invasive diagnostic tools, but their role in adolescents remains investigational and limited by the need for external validation, adolescent-specific validation, accessibility, cost, regulatory clarification, and integration into clinical pathways [13,38]. Non-invasive biomarkers should not replace clinical evaluation or imaging [12,13,38]. At present, their role in adolescents should be considered complementary and exploratory rather than routine. Any biomarker-based approach should be interpreted together with symptoms, functional impairment, imaging findings, patient preference, and response to treatment [7,12,13].

### 3.5. Early Recognition and Function-Preserving Management

The traditional approach to endometriosis has often been reactive: symptoms are treated intermittently, diagnosis is delayed, and structured intervention is introduced only after disease becomes clearly visible on imaging, surgically confirmed, advanced, or fertility-relevant [2,10,12,19]. In adolescents, such delay may be particularly harmful from a clinical and functional perspective, because severe dysmenorrhea, recurrent pelvic pain, school absenteeism, sleep disruption, and repeated inflammatory menstrual episodes may persist for years before an integrated management plan is established [2,5,6,7,19,20,27].

A prevention-oriented approach should not be interpreted as overtreatment of all adolescents with dysmenorrhea [6,7,10,12]. Rather, it supports earlier clinical recognition and individualized care for adolescents with severe, persistent, recurrent, or functionally disabling symptoms, especially when family history, poor response to first-line therapy, imaging findings, or obstructive anomalies raise suspicion of endometriosis or adenomyosis [6,7,12,21,22,26,52]. In this context, hormonal suppression of ovulatory menstruation may be considered a clinically plausible strategy to reduce pain, bleeding, and repetitive menstrual-inflammatory stimulation; however, current adolescent-specific evidence is insufficient to conclude that this approach definitively prevents anatomical disease progression, preserves fertility, or avoids future reproductive harm [2,6,7,10,12].

Adolescents, families, clinicians, and school health professionals should be informed that menstrual pain causing functional impairment should not be normalized as a routine developmental event [5,6,7,20,27]. They should also receive balanced counselling regarding the expected benefits and limitations of hormonal suppression. Appropriately selected medical therapy is generally reversible after discontinuation and may improve symptom control and daily functioning, but its long-term effects on lesion progression, fertility, and obstetric outcomes in adolescents remain uncertain and require prospective longitudinal evaluation [6,7,10,12]. Safety counselling should be individualized, with particular attention to bone health, venous thromboembolism risk, contraindications to estrogen-containing therapy, treatment tolerability, adherence, patient preferences, and quality-of-life outcomes [40,41,42,43,44,45,46].

#### 3.5.1. Medical Treatment

In adolescents with suspected or confirmed endometriosis, treatment usually begins with a medical approach, while surgery is reserved for cases with a clear clinical or imaging-based indication [7,8,12]. Management should focus on reducing dysmenorrhea, cyclic or non-cyclic pelvic pain, abnormal bleeding, analgesic need, and functional limitation, while also integrating safety and acceptability factors such as contraindications, tolerability, adherence, bone health, patient preference, and quality of life [7,8,12,40,41]. Continuous or extended estrogen–progestogen regimens and progestogen-only therapies are generally used as initial hormonal options [7,8,12,47,48]. GnRH agonists, and more cautiously GnRH antagonists, should usually be considered only when symptoms persist despite adequate first-line treatment, when standard hormonal options are not tolerated or contraindicated, or when complex cases require specialist management [7,8,12,48,49]. Possible long-term effects on anatomical progression or fertility remain hypotheses that require prospective studies focused on adolescent populations [2,7,10,12].

The risk–benefit profile of hormonal treatments, focused on counselling considerations rather than graded recommendations, is summarized in Table 5.

#### 3.5.2. Surgery

The available literature provides limited and heterogeneous guidance on the optimal surgical management of endometriosis in adolescent patients, reflecting differences in diagnostic criteria, lesion phenotype, surgical expertise, imaging protocols, and follow-up duration [2,3,12,52,53]. Although endometriosis may progress over time, the choice between early surgical intervention and initial medical suppression remains debated, particularly in adolescents with severe symptoms but no clearly defined surgical target on imaging [2,10,12,14,22,52,53]. When pelvic imaging does not identify large endometriomas, deep infiltrating endometriosis, obstructive anomalies, adnexal masses or organ-threatening disease, surgical evaluation should generally be reserved for selected patients with persistent symptoms despite adequate medical and supportive therapy, contraindications or intolerance to hormonal suppression, unacceptable symptoms despite treatment, diagnostic uncertainty after appropriate counselling, or patient preference after shared decision-making [2,3,7,10,12,14,52,53].

When surgery is performed, laparoscopy should be planned with a therapeutic objective whenever feasible rather than used only to confirm the diagnosis. It should be undertaken by clinicians familiar with adolescent endometriosis, subtle lesion morphology, ovarian-sparing principles, and reproductive-health considerations [3,12,53]. Careful preoperative mapping with expert ultrasound and/or magnetic resonance imaging may support surgical planning and reduce unnecessary diagnostic procedures [2,3,11,12,13,14,15,16].

In adolescents, disease is frequently identified at earlier stages, with superficial peritoneal lesions predominating, whereas advanced stage III–IV disease, deep infiltrating endometriosis and large endometriomas appear less common than in older adult cohorts [2,11,14,22,53]. Nevertheless, early-stage disease can be highly symptomatic, and symptom severity should not be dismissed solely because lesions are small, superficial or not detected on imaging [7,11,13,14,19].

Surgical techniques include ablation, using electrosurgical or laser-based destruction of visible implants, and excision, in which lesions and adjacent fibrotic tissue are removed [53]. In more extensive disease, radical excision may be required to treat deep infiltrating lesions or organ involvement, but such procedures require careful multidisciplinary planning and should be reserved for appropriately selected cases [11,12,53]. In the presence of endometriomas, conservative and ovarian-sparing decision-making is essential because ovarian surgery, particularly repeated or bilateral procedures, may compromise ovarian reserve [3,12,53]. Postoperative medical therapy should be framed mainly around reduction of symptomatic recurrence and longitudinal management; fertility preservation remains a plausible clinical objective but is not fully demonstrated by adolescent-specific prospective evidence [9,12,53].

## 4. Limitations

This narrative review has several limitations. First, the literature search was conducted in a single database, PubMed/MEDLINE, and relevant studies indexed only in Embase, Scopus, Web of Science, CINAHL, or the Cochrane Library may have been missed. Second, the search was restricted to English-language publications, which may have introduced language bias.

Third, no formal methodological quality assessment, risk-of-bias assessment, or evidence grading was performed. Consequently, the findings should be interpreted as a narrative synthesis rather than as graded clinical recommendations. Fourth, the included literature was heterogeneous with regard to study design, age groups, diagnostic criteria, imaging protocols, treatment exposure, and outcome reporting.

Fifth, adolescent-specific prospective evidence remains limited. Some safety, pathophysiological, imaging, biomarker and treatment considerations were therefore informed by adult or mixed-age studies and interpreted cautiously as contextual evidence. Finally, longitudinal evidence regarding anatomical progression, fertility outcomes, obstetric consequences, and the disease-modifying effect of early hormonal suppression in adolescents remains insufficient.

## 5. Conclusions

Adolescent endometriosis should be considered a clinically relevant condition that may begin early in reproductive life, rather than a disease exclusively associated with adult women. Direct adolescent evidence supports early suspicion when severe dysmenorrhea, chronic or acyclic pelvic pain, school absenteeism, impaired daily functioning, poor response to first-line therapy, family history, or obstructive Müllerian anomalies are present. Adult or mixed-age evidence was used only as contextual support when adolescent-specific data were limited.

The clinical approach should emphasize early recognition, non-normalization of disabling menstrual pain, timely referral, expert imaging when appropriate, individualized medical therapy, shared decision-making, and selective surgery. Normal ultrasound or MRI does not exclude superficial or early disease, and escalation of evaluation should consider functional impact, persistence, recurrence, and response to treatment, not imaging findings alone.

Hormonal suppression may reduce dysmenorrhea, cyclic or acyclic pelvic pain, menstrual bleeding, and functional impairment in adolescents. It may also represent a clinically plausible strategy to reduce repetitive ovulatory-menstrual inflammatory stimulation; however, current adolescent-specific prospective evidence is insufficient to conclude that hormonal suppression prevents anatomical progression, preserves fertility, or avoids future reproductive or obstetric complications. Surgery should remain selective, primarily therapeutic, conservative, ovarian-sparing, and imaging-informed. Future research should prioritize prospective adolescent-specific studies evaluating pain trajectories, quality of life, imaging algorithms, biomarkers, treatment safety, recurrence, disease progression, and long-term reproductive outcomes.

## Figures and Tables

**Table 1:** PubMed/MEDLINE search strategy.

Item	Search Details
Database	PubMed/MEDLINE
Final search date	9 August 2026
Core disease terms	(“Endometriosis”[Mesh] OR endometriosis[Title/Abstract])
Age/population terms	(“Adolescent”[Mesh] OR adolescent*[Title/Abstract] OR teenager*[Title/Abstract] OR teenage[Title/Abstract] OR postmenarchal[Title/Abstract] OR “young women”[Title/Abstract] OR “young female”[Title/Abstract])
Symptom and diagnosis terms	dysmenorrhea OR secondary dysmenorrhea OR chronic pelvic pain OR acyclic pelvic pain OR school absenteeism OR quality of life OR ultrasound OR sonography OR transvaginal ultrasound OR MRI OR magnetic resonance imaging OR non-invasive diagnosis OR adenomyosis OR Müllerian anomalies OR obstructive anomalies OR genital tract obstruction
Treatment, safety, and mechanism terms	combined oral contraceptives OR combined hormonal contraceptives OR progestin* OR dienogest OR levonorgestrel intrauterine system OR GnRH agonist OR GnRH antagonist OR add-back therapy OR central sensitization OR bone health OR bone mineral density OR venous thromboembolism OR biomarkers OR salivary microRNA
Search logic	Disease terms AND age/population terms AND symptom/diagnosis/treatment/safety/mechanism terms. Studies retrieved through broader mixed-age terms were included only when relevant to adolescent care.

**Table 2:** Thematic evidence map of the included publications.

Evidence Domain	Included References	Type of Evidence	Population or Focus	Principal Contribution to the Review
General adolescent endometriosis background	[1,2,3,4,5,6,7,8,9,19,20,21]	Book chapter, narrative reviews, expert reviews, clinical reviews, guideline-based documents	Adolescents and young women with suspected or confirmed endometriosis	Provided the clinical framework for adolescent endometriosis, including symptom patterns, diagnostic delay, clinical suspicion, management principles, and adolescent-specific considerations.
Epidemiology and disease burden	[22,23,24]	Epidemiological reviews, clinical reviews, population-level discussion	General endometriosis populations, with adolescent interpretation only when clinically relevant	Supported the discussion of endometriosis prevalence, under-recognition, diagnostic delay, healthcare burden, and comorbidity. These sources were interpreted as contextual evidence rather than adolescent-specific evidence.
Symptom onset, dysmenorrhea, and chronic pelvic pain	[5,6,7,8,17,18,25]	Guidelines, adolescent-focused reviews, observational studies, clinical reviews	Adolescents and young women with dysmenorrhea, pelvic pain, or suspected endometriosis	Supported the relevance of early severe dysmenorrhea, chronic or acyclic pelvic pain, school absenteeism, impaired functioning, and poor response to first-line analgesic therapy as adolescent-relevant warning signs.
Risk factors and protective associations	[5,6,7,8,17,18,20,21,26,27,28,29,30,31,32]	Reviews, systematic reviews, observational studies, meta-analyses, randomized trials	Adolescents, young women, and selected mixed-age populations	Informed the adolescent risk framework, including early menarche, severe dysmenorrhea, family history, obstructive Müllerian anomalies, low BMI, early-life factors, hormonal exposure, and selected lifestyle or nutritional factors. Adult or mixed-age evidence was interpreted with caution and not treated as equivalent to adolescent-specific evidence.
Obstructive Müllerian anomalies and anatomical risk	[28,29]	Observational studies and reviews	Adolescents or young patients with obstructive reproductive tract anomalies	Supported the inclusion of obstructive Müllerian anomalies as adolescent-relevant anatomical risk factors and highlighted the possible role of impaired menstrual outflow and retrograde menstruation.
Imaging and non-invasive diagnosis	[11,13,14,15,33]	Systematic review, imaging studies, clinical studies, diagnostic validation study, narrative review	Adolescents, young women, and patients undergoing non-invasive diagnostic assessment	Supported the role and limitations of ultrasound, MRI, and emerging non-invasive diagnostic tools. Adolescent imaging evidence was prioritized, while biomarker evidence was interpreted cautiously because adolescent-specific validation remains limited.
Deep endometriosis, adenomyosis, and early disease phenotype	[11,14,15,16,33,34]	Imaging studies, clinical studies, mechanistic review	Adolescents and young women with suspected deep endometriosis, adenomyosis, or severe dysmenorrhea	Supported the discussion of early lesion phenotype, deep endometriosis, adenomyosis-related findings, and possible coexistence of endometriosis and early myometrial changes in symptomatic adolescents.
Repetitive ovulatory menstruation and inflammatory pathophysiology	[10,34,35,36]	Mechanistic reviews, conceptual reviews, clinical hypothesis papers	Early-onset endometriosis, adenomyosis, and general reproductive-age pathophysiology	Supported the biological plausibility of a model linking ovulation, menstruation, inflammation, estrogen exposure, tissue remodeling, endometriosis, and adenomyosis. These concepts were presented as hypotheses or plausible mechanisms rather than definitive adolescent-specific proof of disease progression.
Central sensitization, recurrent pain, psychological burden, and quality of life	[6,8,9,17,18,25,37]	Adolescent reviews, observational studies, clinical reviews, mixed-methods study	Adolescents, young women with dysmenorrhea, chronic pelvic pain, or endometriosis-related symptoms	Supported the discussion of recurrent pain, transition from cyclic to acyclic pain, school absenteeism, impaired daily functioning, psychological burden, and the need for function-based follow-up. Adult or mixed-age patient-experience data were used only as contextual evidence.
Non-invasive biomarkers and salivary microRNA	[13,38,39]	Systematic review, diagnostic validation study, narrative review	Patients with suspected or confirmed endometriosis; adolescent applicability interpreted cautiously	Supported the discussion of salivary microRNA signatures and other non-invasive diagnostic approaches. Their role in adolescents was presented cautiously because age-specific validation remains limited.
Medical treatment: general endometriosis management	[3,6,7,8,9,10,12,35,40,41]	Guidelines, reviews, expert consensus, eligibility guidance, conceptual review	Adolescents, young women, and selected mixed-age populations	Supported first-line and second-line treatment classification, empirical medical management, hormonal suppression, treatment eligibility assessment, and individualized counselling. Guideline and adult/mixed-age evidence were interpreted according to their level of applicability to adolescent care.
Combined hormonal contraception and adolescent safety	[7,38,41,42,43,44,45]	Guideline-based evidence, systematic reviews, meta-analysis, adolescent safety reviews	Adolescents using combined hormonal contraception or requiring contraceptive eligibility assessment	Supported counselling regarding combined hormonal contraception, contraindications, eligibility assessment, and bone-health considerations during adolescence. Adult-only cancer-risk data were not used as central adolescent treatment evidence.
Progestin therapy, dienogest, LNG-IUS, and adolescent tolerability	[7,12,40,41,46,47,48,49,50,51]	Guidelines, systematic reviews, adolescent treatment study, adolescent safety studies, narrative review	Adolescents and young women receiving progestin-based therapy, dienogest, LNG-IUS, or long-acting reversible contraception	Supported the use of progestin-based treatment, dienogest, LNG-IUS, adherence counselling, tolerability, thrombotic safety considerations, bone-health issues, and adolescent-specific counselling. Mixed-age safety data were used cautiously when adolescent-specific evidence was limited.
GnRH agonists and add-back therapy	[7,8,36,39,48,49]	Guideline-based evidence, clinical review, randomized trial, systematic review/meta-analysis, adolescent bone-density study	Adolescents and young women requiring second-line therapy	Supported the role of GnRH agonists as second-line treatment, the need for add-back therapy, and bone-health monitoring in adolescents. Evidence regarding long-term disease modification or fertility preservation was not considered definitive.
GnRH antagonists	[12,36]	Guideline-based evidence and systematic review/meta-analysis	Adolescent use interpreted cautiously	Supported cautious discussion of GnRH antagonists as potential second-line options. Because adolescent-specific evidence is limited, these agents were presented as specialist-level options rather than routine adolescent therapy.
Surgery and surgical decision-making	[2,3,7,9,11,12,16,52,53]	Guidelines, adolescent cohort, imaging studies, expert commentary, adolescent-focused reviews	Adolescents and young women with suspected or confirmed endometriosis	Supported selective, primarily therapeutic, conservative, and ovarian-sparing surgery. The evidence also supported imaging-informed surgical planning and postoperative medical management when appropriate.
Fertility, ovarian reserve, and long-term reproductive considerations	[2,3,7,9,10,12,52,53]	Guidelines, adolescent reviews, cohort evidence, conceptual review, expert commentary	Adolescents and young women with suspected or confirmed endometriosis, endometriomas, or surgical indication	Supported cautious counselling regarding fertility, ovarian reserve, and long-term reproductive considerations. Claims that hormonal suppression prevents progression, preserves fertility, or avoids future reproductive harm were moderated because prospective adolescent-specific evidence remains insufficient.

Note: Publications were classified as adolescent-specific when they directly addressed adolescents, teenage patients, postmenarchal girls, or adolescent/young female cohorts with direct relevance to adolescent care. Adult or mixed-age evidence was retained only when adolescent-specific evidence was limited and when the information was clinically relevant to adolescent management, particularly for guideline recommendations, treatment safety, bone health, venous thromboembolism risk, pathophysiology, or non-invasive diagnostic concepts. These sources were interpreted as contextual evidence and were not considered equivalent to adolescent-specific prospective data.

**Table 3:** Clinically actionable adolescent warning signs for suspected endometriosis.

Evidence Category	Clinical Feature	Interpretation in Adolescents	References
Clinical red flag	Severe dysmenorrhea beginning at or shortly after menarche	Marked early menstrual pain should prompt suspicion, especially when progressive or functionally disabling.	[5,6,7,8]
Clinical red flag	Persistent pain despite adequate NSAIDs and/or hormonal treatment	Insufficient response to appropriate first-line therapy should prompt evaluation for secondary dysmenorrhea.	[7,8,18]
Clinical red flag	Dysmenorrhea associated with acyclic pelvic pain	Coexistence of cyclic and non-cyclic pain may indicate a chronic pelvic pain phenotype.	[6,7,8]
Functional red flag	School absenteeism, sleep disruption, reduced sport participation, or limitation of daily activities	Functional impairment should influence referral or escalation even when imaging is negative.	[6,7,17]
Familial/anatomical risk	Positive family history of endometriosis	Family history should lower the threshold for suspicion in adolescents with severe symptoms.	[5,6,20,21]
Anatomical risk	Obstructive Müllerian anomalies or genital outflow obstruction	Obstructive anomalies are particularly relevant because impaired menstrual outflow may favor retrograde menstruation.	[28,29]

**Table 4:** Contextual risk factors and associations requiring cautious interpretation in adolescents.

Evidence Category	Factor or Association	Interpretation in Adolescents	References
Epidemiological association	Early menarche	May increase cumulative menstrual exposure, but should not be used alone as a clinical predictor.	[5,21,22,26]
Epidemiological association	Short menstrual cycles or heavy/prolonged bleeding	May strengthen suspicion when combined with severe pain or functional impairment.	[6,8,20,22,23,26]
Contextual association	Low BMI or lean body habitus	Reported in association with endometriosis, but weak as an isolated adolescent predictor.	[22,23,27]
Contextual association	Prematurity or low birth weight	Early-life associations are reported, but individual predictive value in adolescents remains limited.	[30]
Contextual association	Asthma or atopic disease	Reported in adolescent/young adult populations but weaker than menstrual, familial, or obstructive red flags.	[20]

Note: Table 3 prioritizes clinically actionable adolescent warning signs, whereas Table 4 summarizes contextual or weaker associations. These associations should not be interpreted as representing equivalent levels of evidence.

**Table 5:** Counselling considerations for hormonal therapies used for endometriosis-associated pain in adolescents.

Treatment Class	Adolescent-Relevant Benefits	Adolescent-Relevant Risks, Limitations and Counselling Points
Combined oral contraceptives/combined hormonal contraceptives COC/CHC	Continuous or extended combined estrogen–progestogen regimens may reduce dysmenorrhea, menstrual bleeding, withdrawal bleeding, and cyclic pelvic pain, supporting school attendance, sleep, physical activity, and daily functioning [7,8,12]. CHC is reversible, accessible, and may be used as empirical first-line medical management after contraindications are excluded [7,8,12,40,41].	Breakthrough bleeding, nausea, breast tenderness, headache, mood symptoms, and adherence difficulties should be discussed [7,8,40]. Estrogen-containing therapy requires screening for thrombotic history, thrombophilia, migraine with aura, severe hypertension, prolonged immobilization, and other major vascular risk factors [40,41]. Bone-health considerations remain relevant during prolonged adolescent use [42,43,44,45].
Progestins/levonorgestrel-releasing intrauterine system Progestogen therapy/LNG-IUS	Progestin-based therapies may reduce dysmenorrhea, cyclic pelvic pain, and bleeding. Dienogest has adolescent-specific evidence supporting its use for clinically suspected endometriosis-associated pain [7,8,12,47,50]. Progestin-only therapy is useful when estrogen is contraindicated or undesirable [40,41]. LNG-IUS may support prolonged bleeding control when acceptable to the patient [7,40,51].	Irregular bleeding, spotting, acne, mood symptoms, breast tenderness, and perceived weight change may affect adherence [7,8,40,47]. Dienogest may be associated with modest lumbar spine BMD reduction, while depot medroxyprogesterone acetate is more consistently associated with BMD loss during prolonged use [43,45,47,48]. LNG-IUS acceptability should be individualized through shared decision-making [7,40,51].
GnRH agonists	GnRH agonists may be considered for persistent pain despite first-line therapy, intolerance or contraindications to CHC/progestins, or selected complex cases requiring specialist management [7,8,12,48,49]. Add-back therapy may improve tolerability and reduce hypoestrogenic bone loss [48,49].	Hypoestrogenism may cause vasomotor symptoms, vaginal dryness, sleep disturbance, mood changes, and BMD loss, especcially during peak bone mass acquisition [7,8,45,48,49]. Treatment should generally be time-limited, combined with add-back therapy when appropriate, and monitored for response, adverse effects, adherence, and bone health [7,8,48,49]. combined with add-back therapy when appropriate, and monitored for response, adverse effects, adherence, and bone health [7,8,12,48].
GnRH antagonists	Oral GnRH antagonists provide rapid and reversible ovarian suppression, but adolescent-specific evidence remains limited [12]. Their use should remain within specialist care after careful assessment of indications, risks, and alternatives [12,40,41].	Use should be cautious, individualized, and aligned with regulatory indications and specialist guidance [12,40,41]. Dose-dependent estrogen suppression may affect BMD and may cause hypoestrogenic symptoms [12,45]. Age-related approval status, cost, availability, adherence, tolerability, and patient preference should be considered [12,40,41].
Shared considerations across all hormonal therapies	Hormonal treatment should primarily aim to reduce dysmenorrhea, cyclic or acyclic pelvic pain, menstrual bleeding, analgesic use, school absenteeism, and impairment in daily functioning [7,8,12]. Treatment choice should be guided by symptom severity, bleeding profile, contraindications, bone-health profile, need for contraception, tolerability, adherence, patient preference, emotional readiness, and quality-of-life impact [7,8,40,41].	Current adolescent-specific evidence does not permit definitive conclusions about whether hormonal suppression prevents anatomical disease progression, preserves fertility, or avoids future reproductive harm. These potential benefits remain clinically plausible but unproven [2,7,10,12]. Follow-up should assess pain, bleeding pattern, medication adherence, side effects, school attendance, sleep, physical activity, psychological wellbeing, and the need for treatment adjustment or specialist referral [7,8,12].

Note: This table summarizes counselling considerations for adolescents and does not provide formal hierarchical recommendations based on graded evidence quality. Treatment decisions should follow current guidelines and individualized specialist assessment. Abbreviations: BMD, bone mineral density; CHC, combined hormonal contraceptive; COC, combined oral contraceptive; GnRH, gonadotropin-releasing hormone; LNG-IUS, levonorgestrel-releasing intrauterine system; NSAIDs, non-steroidal anti-inflammatory drugs; VTE, venous thromboembolism.

## Data Availability

No new data were created or analyzed in this study. Data sharing is not applicable to this article.

## Abbreviations

The following abbreviations are used in this manuscript:

BMD	Bone mineral density
BMI	Body mass index
CHC	Combined hormonal contraceptive
COC	Combined oral contraceptive
GnRH	Gonadotropin-releasing hormone
LNG-IUS	Levonorgestrel-releasing intrauterine system
MRI	Magnetic resonance imaging
miRNA	MicroRNA
NSAIDs	Non-steroidal anti-inflammatory drugs
VTE	Venous thromboembolism
